# The Role of Honey in Dental Caries Prevention: A Narrative Review

**DOI:** 10.7759/cureus.106948

**Published:** 2026-04-13

**Authors:** Pierluigi Valente, Lapo Sbrenna, Andrea Mascolo, Andrea Sbrenna, Francesco Valente

**Affiliations:** 1 Dentistry, Vita-Salute San Raffaele University, Milan, ITA; 2 Dentistry, Alma Mater Studiorum - University of Bologna, Bologna, ITA; 3 Academics, European Institute for Medical Studies, St. Julian's, MLT; 4 Dentistry, Humanis Dental Center, Perugia, ITA; 5 Oral Implantology, San Damiano Dental Clinic, Rome, ITA

**Keywords:** antibacterial activity, antibiofilm effect, dental caries, honey, oral microbiota, preventive dentistry, streptococcus mutans

## Abstract

Dental caries is a multifactorial infectious disease influenced by diet and dental biofilm activity, characterized by acid production from bacterial fermentation of dietary carbohydrates, with *Streptococcus mutans* playing a central role in its development. It remains one of the most prevalent noncommunicable diseases worldwide. In recent years, honey has emerged as a natural agent of interest in oral healthcare due to its antibacterial, antibiofilm, anti-inflammatory, and wound-healing properties. In vitro studies indicate that honey exerts a multifactorial antimicrobial effect mediated by hydrogen peroxide, polyphenols, bioactive peptides, and a complex mixture of over 200 compounds, effectively inhibiting bacterial growth and biofilm formation. Its activity has been demonstrated against key oral pathogens, including *S. mutans* and *Lactobacillus* species, although potency varies depending on honey type, concentration, and formulation. Clinical evidence suggests that honey may reduce plaque accumulation and improve gingival health, but its effectiveness generally remains inferior to conventional agents such as chlorhexidine. Novel honey-based formulations, including toothpastes and mouth rinses, show promising antibiofilm activity; however, clinical validation remains limited. In addition, the effects of honey on enamel integrity remain unclear, with in vitro studies reporting heterogeneous findings, ranging from potential protective or neutral outcomes to possible demineralizing effects depending on experimental conditions. Overall, honey may serve as a complementary adjunct in oral hygiene and caries prevention but cannot replace established preventive measures. Well-designed, long-term clinical trials with standardized formulations are needed to confirm its efficacy and establish practical guidelines for its use in dental care.

## Introduction and background

Dental caries is a biofilm-mediated, diet-modulated, multifactorial disease characterized by progressive demineralization of dental hard tissues driven by acid production from bacterial fermentation of dietary carbohydrates. Among cariogenic microorganisms, *Streptococcus mutans* plays a central role due to its acidogenicity, aciduricity, and ability to form structured biofilms. It represents a major global public health issue and the most prevalent noncommunicable disease worldwide, affecting both permanent and primary teeth.

An estimated two billion adults and 510 million children are affected globally [1]. High intake of free sugars is a primary risk factor, contributing not only to caries but also to overweight and obesity, underscoring the need for simple, cost-effective preventive strategies at the population level. Early, minimally invasive interventions can be implemented in primary care without specialized equipment. Untreated caries can lead to significant morbidity, including tooth loss, pain, functional impairments (e.g., eating and speaking), and broader impacts on quality of life and psychosocial well-being. According to the Global Burden of Disease 2021, untreated caries in permanent teeth is the most common health condition worldwide. Recent estimates indicate that oral disorders affected approximately 3.74 billion people in 2021, with caries in permanent teeth increasing by 6% and periodontal disease incidence rising by over 76% [2,3].

Given the global burden of dental caries and the limitations of conventional preventive measures, there is growing interest in complementary natural strategies, including honey, for plaque control and caries prevention. Dental caries is a multifactorial infectious disease primarily driven by acidogenic and aciduric bacteria, particularly *S. mutans*, which plays a central role in biofilm formation and enamel demineralization [4,5]. Conventional preventive strategies combine mechanical plaque control with chemical agents, such as fluoride and chlorhexidine; however, these approaches may be limited by side effects or suboptimal patient compliance [6,7]. In this context, natural products have attracted attention as potential adjuncts or alternatives in oral healthcare [6].

Honey, produced by *Apis mellifera*, contains over 200 bioactive compounds, including sugars, organic acids, enzymes, and polyphenols, whose composition varies with botanical origin [8]. Historically used in wound care, honey exhibits broad-spectrum antimicrobial activity, prompting investigation into its application in dentistry for the prevention and management of conditions such as caries, gingivitis, and mucositis [4,9].

The rationale for its use in cariology lies in its complex antibacterial mechanisms, including osmotic effects, low pH, hydrogen peroxide production, and antimicrobial peptides [5,10]. However, despite promising in vitro findings, the translation of these effects into clinical practice remains uncertain due to variability in study designs, honey composition, and treatment protocols [4].

Aim

This narrative review aims to provide a comprehensive overview of the antimicrobial and antibiofilm properties of honey, with particular emphasis on its effects against cariogenic microorganisms involved in dental biofilm formation. In addition, the review seeks to critically evaluate the current evidence regarding the potential role of honey as a complementary strategy in preventive dentistry. By integrating findings from in vitro, in vivo, and clinical studies, this work aims to clarify the mechanisms of action of honey and assess its applicability in caries prevention and oral health management, while highlighting existing limitations and areas for future research.

## Review

Methods

Search Strategy

The literature included in this review was retrieved through electronic searches in PubMed and Scopus databases, focusing on studies published from 2012 to January 2026. The search strategy was developed using Boolean operators to combine relevant keywords, including (“honey” OR “Manuka honey”) AND (“oral health” OR “dental caries” OR “oral microbiota”) AND (“antibacterial activity” OR “antimicrobial” OR “biofilm control”). Studies were selected based on their conceptual relevance to the effects of honey on cariogenic bacteria, plaque formation, and oral microbiota, with particular emphasis on in vitro, in vivo, and clinical investigations. Additional manual screening of reference lists was performed to identify further relevant studies. Given the narrative nature of this review, the study does not adhere to the PRISMA guidelines and does not include a formal risk of bias assessment. This represents an inherent limitation, as the methodology does not allow for systematic quantitative comparison of outcomes or formal minimization of selection bias. However, the narrative approach was intentionally adopted to enable a broader synthesis and critical interpretation of the available evidence, integrating mechanistic, microbiological, and clinical perspectives on the role of honey in oral health.

Discussion

Antibacterial Mechanisms of Honey

A growing body of in vitro evidence supports the antibacterial and antibiofilm activity of honey against cariogenic microorganisms [4]. Several studies have demonstrated that honey can inhibit the growth of* S. mutans*, a key pathogen in the initiation and progression of dental caries [5,11]. In particular, different honey varieties have shown variable antibacterial efficacy, with buckwheat, honeydew, and Manuka honey exhibiting the strongest inhibitory activity against *S. mutans* [5]. This antimicrobial effect appears to be multifactorial and largely attributed to hydrogen peroxide production, enzymatic activity (e.g., glucose oxidase), high osmolarity, and the presence of polyphenols and other bioactive compounds. Furthermore, experimental findings indicate that enzymatic degradation of hydrogen peroxide significantly reduces the antibacterial activity of honey, supporting its central role in mediating antimicrobial effects [5].

Recent research has also highlighted the role of extracellular vesicles derived from honey, which act as carriers of bioactive molecules with antimicrobial properties. These honey-derived exosome-like extracellular vesicles have been shown to contain antimicrobial peptides such as defensin-1, jellein-3, and MRJP1, contributing to bacterial membrane disruption and biofilm inhibition [10]. Experimental findings demonstrated that these vesicles exert a differential antibacterial effect against oral streptococci, with a more pronounced activity against *S. mutans* compared to *Streptococcus sanguinis* and induce nanomechanical alterations consistent with membrane damage [10]. These observations suggest a novel mechanism by which honey may modulate biofilm formation at the molecular level. Additionally, the antibacterial efficacy of honey varies significantly depending on its botanical origin and physicochemical composition. In particular, Manuka honey has been shown to exhibit enhanced antimicrobial activity due to its non-peroxide activity (NPA), which represents an additional antibacterial mechanism independent of hydrogen peroxide [12]. Notably, an NPA-dependent effect has been reported, with higher NPA levels (≥15) associated with significantly greater antibacterial activity against oral pathogens. However, this activity appears to be species-dependent, with stronger inhibitory effects observed against *Porphyromonas gingivalis* and *Aggregatibacter actinomycetemcomitans* than against *S. mutans* [12].

From a biofilm perspective, honey-based formulations have demonstrated the ability to significantly reduce bacterial viability and metabolic activity within multispecies biofilms, although their impact on the structural integrity of established biofilm mass appears limited [13]. In particular, in vitro data showed that Manuka honey-containing toothpastes were associated with a reduction in colony-forming units (CFUs) and metabolic activity in preformed biofilms, with reductions of up to approximately 2.3 log₁₀ CFU at higher concentrations, while no significant decrease in total biofilm mass was observed [13]. Furthermore, when applied prior to bacterial colonization, these formulations demonstrated a concentration-dependent inhibition of de novo biofilm formation, suggesting a preventive rather than disruptive effect on biofilm development [13]. These findings indicate that honey-containing products may be more effective in limiting bacterial proliferation and early biofilm establishment rather than eradicating mature, structurally organized biofilms.

Clinical Evidence and Limitations

Clinical studies provide more heterogeneous results. Some randomized controlled trials have demonstrated that honey can reduce plaque accumulation, bacterial counts, and gingival inflammation, supporting its potential role as an adjunctive oral hygiene agent [14,15]. In orthodontic patients, honey has been shown to significantly reduce the counts of *S. mutans*, lactobacilli, and* P. gingivalis* with antibacterial effects that were statistically superior to control solutions and comparable or even greater than those observed with some conventional antimicrobial agents [14]. Importantly, despite containing fermentable sugars, honey demonstrated a more favorable effect on plaque pH dynamics compared to sucrose. Although an initial pH drop was observed within the first minutes after exposure, plaque pH rapidly recovered within 10-20 minutes and did not fall below the critical threshold for enamel demineralization (pH 5.5), unlike sucrose, which induced a more pronounced and prolonged acidification [14]. This finding suggests a potentially lower cariogenic risk profile compared to conventional dietary sugars. In addition, randomized controlled trials evaluating honey-based mouthwashes have reported significant reductions in plaque and gingival indices over short-term follow-up periods, with effects comparable between different types of honey, including Manuka and raw honey [15]. However, chlorhexidine consistently demonstrated superior efficacy in reducing clinical parameters, highlighting that while honey may represent a promising natural adjunct, it does not yet match the effectiveness of established chemical agents. When compared with conventional agents such as chlorhexidine, honey generally exhibits lower antimicrobial efficacy in reducing plaque and gingival indices [7,15]. In a randomized controlled field trial with a 21-day follow-up, honey-based mouthwashes (both Manuka and raw honey) resulted in significant reductions in plaque and gingival scores; however, chlorhexidine consistently demonstrated the greatest improvement in clinical parameters, confirming its superior antiplaque and antigingivitis efficacy [15].

Similarly, a randomized pilot clinical trial evaluating stingless bee honey mouth rinse under conditions without mechanical oral hygiene reported no significant reduction in plaque accumulation compared to baseline or saline controls over a three-day period, whereas chlorhexidine produced a statistically significant decrease in plaque scores [7]. These findings suggest that the antiplaque effect of honey may be insufficient when used as a standalone intervention, particularly in the absence of mechanical plaque control. Nevertheless, honey-based formulations demonstrated favorable tolerability profiles, with significantly better patient-reported outcomes in terms of taste and reduced burning sensation compared to chlorhexidine, which may enhance patient compliance in long-term use [7]. Taken together, these data indicate that while honey may represent a promising natural adjunct in oral hygiene, its clinical efficacy remains inferior to gold standard antiseptics, particularly in plaque control, and appears to be strongly dependent on its use in combination with mechanical oral hygiene practices [7,15].

Effects on Enamel and Demineralization

In addition to antimicrobial effects, honey has also been investigated for its potential role in remineralization strategies. In vitro studies using demineralized enamel specimens have shown that honey-based formulations, such as honey-ginger paste, can improve surface microhardness and reduce surface roughness following pH-cycling protocols simulating cariogenic challenges [16]. Specifically, after a 21-day treatment period, enamel treated with honey-based formulations demonstrated microhardness values comparable to those achieved with fluoride-containing toothpaste, with similar reductions in surface roughness, suggesting a partial recovery of enamel structural integrity. Notably, no statistically significant differences were observed among the tested remineralizing agents, including fluoride, honey-based paste, and ozone therapy, indicating that honey-containing formulations may provide a remineralization effect comparable, though not superior, to established fluoride-based approaches [16]. These findings suggest that honey may contribute to noninvasive caries management strategies; however, its role should be interpreted with caution, given the in vitro nature of the evidence and the absence of long-term clinical validation.

The effects of honey on enamel integrity remain a subject of ongoing investigation, with contrasting evidence emerging from recent in vitro studies. Safii et al. evaluated the antibacterial properties of medical-grade Manuka honey against a spectrum of oral bacteria, including periodontopathogens such as *P. gingivalis*, *Fusobacterium nucleatum*, and *Prevotella intermedia*. While honey exhibited broad antimicrobial activity, *S. mutans*, a cariogenic species, demonstrated notable resistance. The authors further assessed the demineralizing potential of honey on hydroxyapatite beads, a model for dental hard tissues, and observed a pH-dependent release of calcium. This effect was exacerbated in the presence of* S. mutans*, likely due to the fermentation of honey sugars generating additional acids [17]. These findings indicate that although Manuka honey may offer antimicrobial benefits, its intrinsic acidity and high fermentable carbohydrate content could contribute to enamel and cementum demineralization under prolonged exposure, highlighting a delicate balance between therapeutic efficacy and potential mineralized tissue damage.

In contrast, Habluetzel et al. investigated whether honey and its bioactive components, including methylglyoxal (MGO), hydrogen peroxide, and propolis, affect enamel erosion when applied in the presence of a salivary pellicle. Interestingly, despite the low pH of the tested honeys, no enamel erosion was observed, and pellicle modification with honey or its components did not confer additional protection against acid-induced demineralization. Microbiological assays further demonstrated that honey could inhibit early bacterial colonizers in a species-specific manner, while propolis reduced adhesion of *Streptococcus gordonii *[18]. These findings suggest that the interaction between honey and the enamel surface is modulated by the protective role of the salivary pellicle, which may attenuate the direct demineralizing effects observed in models lacking physiological oral defenses. Overall, the impact of honey on dental hard tissues appears to be highly context-dependent. Factors such as honey type, intrinsic pH, sugar composition, presence of oral microbiota, duration of exposure, and the protective influence of the salivary pellicle collectively determine whether honey exerts a demineralizing, neutral, or potentially protective effect. Although Manuka honey demonstrates notable antimicrobial properties, caution is warranted regarding its direct application to mineralized tissues, particularly under conditions that may promote prolonged acidic exposure or bacterial fermentation. Further studies are required to establish safe and effective approaches for harnessing the therapeutic potential of honey in oral health without compromising enamel and root integrity.

Variability and Lack of Standardization of Honey

An important source of heterogeneity across studies is the lack of standardization of honey products. The antibacterial efficacy of honey varies significantly depending on its botanical origin and physicochemical characteristics, particularly its NPA and MGO content. Standardized grading systems such as the Unique Manuka Factor (UMF) have been proposed to improve comparability across studies.

Honey has been proposed as a safer alternative to synthetic agents due to its lower incidence of adverse effects and better patient acceptability [7,9]. Clinical evidence indicates that honey-based mouth rinses are associated with improved tolerability profiles, with significantly lower reports of burning sensation and more favorable taste perception compared to chlorhexidine, which may positively influence patient compliance during prolonged use [7]. In addition to its favorable safety profile, honey exhibits a broad spectrum of biological activities, including antibacterial, anti-inflammatory, and wound-healing properties, which may contribute synergistically to oral health maintenance and disease management [9]. These multifunctional effects support its potential application in a wide range of oral conditions, including dental caries, gingivitis, halitosis, xerostomia, and mucosal lesions associated with radiotherapy or post-extraction healing [9].

This variability in composition directly contributes to the heterogeneity observed in clinical outcomes, and the clinical efficacy of honey as a primary therapeutic agent is not yet fully established, reinforcing the need for well-designed, long-term randomized controlled trials to validate its safety and effectiveness in routine dental practice [7,9].

To further contextualize the variability observed across different study designs, additional evidence highlights how the antimicrobial efficacy of honey may depend on both formulation and experimental conditions. In an in vitro study, Mathai et al. evaluated the antibacterial activity of honey alone and in combination with other natural extracts against *S. mutans *using standardized culture-based assays [19]. The authors reported that honey alone exhibited relatively limited inhibitory activity, particularly at lower concentrations, when compared to more potent natural antimicrobial agents such as garlic extract. However, when combined with other bioactive substances, honey demonstrated enhanced antibacterial effects, suggesting the presence of potential synergistic interactions. This synergism may be attributed to complementary mechanisms of action, including disruption of bacterial cell membranes, interference with metabolic pathways, and modulation of oxidative stress within microbial cells. Furthermore, the study highlighted that the antimicrobial efficacy of honey is not solely dependent on its sugar content or osmotic properties, but rather on the complex interplay of its bioactive components, which may act more effectively in combination with other natural compounds. These findings support the hypothesis that honey-based formulations could be optimized through combination strategies to enhance antibacterial efficacy, particularly in the context of biofilm-associated oral pathogens.

In contrast, clinical findings suggest that under in vivo conditions, honey-based formulations may achieve more pronounced antibacterial effects. In a randomized controlled trial, Jain et al. evaluated the antimicrobial efficacy of a Manuka honey-based mouthrinse compared with 0.2% chlorhexidine in reducing salivary counts of* S. mutans *and *Lactobacillus acidophilus *over a two-week period [20]. Quantitative microbiological analysis demonstrated a statistically significant reduction in both cariogenic species following the intervention, with no significant differences between the two agents, indicating comparable short-term antimicrobial efficacy under the tested conditions. From a mechanistic perspective, these findings may reflect the multifactorial antibacterial properties of Manuka honey, including the contribution of MGO and other non-peroxide components, which may act synergistically in the complex oral environment. However, it is important to consider that the study duration was limited and did not assess longer-term clinical endpoints such as plaque maturation or caries progression.

These observations further support the concept that the antimicrobial activity of honey is highly context-dependent, varying according to its physicochemical composition, concentration, formulation, and mode of delivery. As highlighted by Deglovic et al. [4], in vitro studies consistently demonstrate antibacterial and antibiofilm activity against cariogenic microorganisms; however, the translation of these effects into clinical settings is influenced by additional factors, including salivary flow, buffering capacity, biofilm complexity, and patient-related variables. Consequently, while laboratory evidence provides strong mechanistic support for the antimicrobial potential of honey, clinical findings remain heterogeneous and sometimes contradictory, limiting the ability to draw definitive conclusions regarding its routine application in caries prevention.

Additional evidence bridging experimental and clinical findings is provided by a randomized controlled study by Aparna et al., which evaluated both the in vitro antibacterial activity and the in vivo antiplaque efficacy of a honey-based mouth rinse. In the in vitro phase, honey demonstrated inhibitory effects against multiple oral bacterial species, as evidenced by measurable antimicrobial activity across tested strains; however, chlorhexidine consistently exhibited lower minimum inhibitory concentrations, indicating superior antimicrobial potency under controlled conditions. In the in vivo component, conducted using a four-day plaque regrowth model, the honey-based mouth rinse was associated with a statistically significant reduction in plaque accumulation compared to saline controls, confirming its biological activity in a clinical setting. Nevertheless, chlorhexidine again demonstrated greater efficacy in inhibiting plaque formation, reinforcing its role as the gold standard antiplaque agent [21].

Importantly, the discrepancy observed between in vitro antimicrobial performance and in vivo clinical efficacy underscores the complexity of the oral environment, where factors such as salivary dynamics, biofilm architecture, and substrate availability may modulate the activity of honey-based formulations. The short duration of the plaque regrowth model further limits the extrapolation of these findings to long-term clinical outcomes. Therefore, while this study provides valuable translational insight by integrating laboratory and clinical data within a single experimental framework, it also highlights the inherent limitations in directly translating in vitro antimicrobial effects into predictable clinical performance. These findings are consistent with the broader body of evidence, suggesting that although honey exhibits measurable antibacterial and antiplaque properties, its clinical effectiveness remains inferior to established antiseptic agents and is strongly influenced by contextual and methodological variables.

Future Perspectives

Future research should prioritize the standardization of honey-based formulations to reduce variability across studies and enhance reproducibility. This includes the systematic characterization and reporting of physicochemical properties such as botanical origin, pH, osmolarity, hydrogen peroxide activity, and non-peroxide components, particularly MGO content and grading systems such as the UMF. Establishing standardized classification criteria will be essential to enable meaningful comparison between studies and to facilitate clinical translation.

From a clinical perspective, there is a need for well-designed randomized controlled trials with larger sample sizes, adequate statistical power, and extended follow-up periods to assess the long-term effects of honey on plaque control, caries incidence, and enamel integrity. Future studies should also incorporate standardized clinical endpoints, including validated plaque and gingival indices, quantitative microbiological assessments, and, where feasible, objective measures of enamel demineralization and remineralization. In addition, comparative effectiveness studies evaluating honey-based formulations against gold standard agents such as chlorhexidine and fluoride-based products would provide clinically relevant insights into their relative performance.

At the mechanistic level, further investigation is warranted to elucidate the molecular pathways underlying the antimicrobial and antibiofilm activity of honey, including the role of bioactive compounds, oxidative stress modulation, and extracellular vesicles. In particular, the contribution of synergistic interactions among honey components, as well as between honey and other natural or conventional antimicrobial agents, represents a promising area for future research.

Finally, given the complexity of the oral ecosystem, future studies should aim to better replicate in vivo conditions by incorporating multispecies biofilm models and considering host-related factors such as salivary flow, buffering capacity, and individual variability. Such approaches may help to bridge the gap between experimental findings and clinical outcomes, ultimately supporting the development of evidence-based guidelines for the safe and effective use of honey in oral healthcare.

Synthesis of Evidence and Tables Overview

To facilitate a structured comparison across studies characterized by heterogeneous designs, experimental models, and outcome measures, key investigations were systematically synthesized into two summary tables. Table 1 provides a focused overview of in vitro studies investigating the effects of honey on enamel mineralization and demineralization, highlighting differences in experimental conditions, substrates, and assessment methods. Table 2 integrates both in vitro and in vivo evidence, summarizing antibacterial, antibiofilm, and clinical outcomes associated with honey-based interventions. This dual-tabulation approach was adopted to improve interpretability, allow cross-study comparison, and provide a clearer distinction between mechanistic findings and clinically relevant evidence, thereby supporting a more comprehensive and critical appraisal of the current literature.

**Table 1:** In vitro studies evaluating the effects of honey on enamel mineralization and demineralization MGO: methylglyoxal; MH: Manuka honey

Study	Study type	Enamel mineralization/demineralization findings
Kade et al. (2022) [16]	In vitro	Honey-ginger paste, fluoride toothpaste, and ozone therapy improved enamel microhardness and reduced surface roughness after 21 days; honey-based formulation comparable to fluoride
Safii et al. (2017) [17]	In vitro	MH caused pH-dependent demineralization of hydroxyapatite; effect enhanced by *Streptococcus mutans* fermentation, indicating a potential risk to enamel and cementum
Habluetzel et al. (2018) [18]	In vitro	Honey and bioactive components (MGO, H₂O₂, propolis) did not induce enamel erosion on salivary-pellicle-covered specimens; pellicle mitigated demineralization

**Table 2:** In vitro and clinical studies evaluating the antibacterial effects of honey on oral pathogens CFU: colony-forming unit; CHX: chlorhexidine; HEc-EVs: honey-derived exosome-like extracellular vesicles; MH: Manuka honey; NPA: non-peroxide activity; OH: oral hygiene; RH: raw honey

Study	Honey/preparation	Bacteria tested	Key findings
Deglovic et al. (2022) [4]	Various honeys	Periodontal pathogens, *Streptococcus mutans*	Consistent antibacterial activity; limited clinical evidence
Grabek-Lejko and Hyrchel (2023) [5]	Various honeys	*S. mutans*	Strong antibacterial activity; H₂O₂ identified as key factor
Reduan et al. (2025) [7]	Stingless bee honey rinse	Plaque	No significant reduction without OH; better tolerability vs CHX
Leiva-Sabadini et al. (2021) [10]	HEc-EVs	*S. mutans*, *Streptococcus sanguinis*	Antibacterial and antibiofilm activity; greater effect on *S. mutans*
Ahmadi-Motamayel et al. (2013) [11]	Natural honey (various concentrations)	*S. mutans*, *Lactobacillus*	Concentration-dependent antibacterial effect; *S. mutans* inhibited at >20%; *Lactobacillus* at higher concentrations
Schmidlin et al. (2014) [12]	MH (variable NPA)	*S. mutans*, *Porphyromonas gingivalis*, *Aggregatibacter actinomycetemcomitans*	NPA-dependent antibacterial effect; stronger activity vs periodontal pathogens
Jungbauer et al. (2025) [13]	MH/propolis toothpaste	Oral bacteria, biofilm	Reduced CFU and metabolic activity; limited effect on biofilm mass
Atwa et al. (2014) [14]	Chewing honey	*S. mutans*, lactobacilli, *P. gingivalis*	Reduced bacterial counts; maintained plaque pH above critical threshold
Singhal et al. (2018) [15]	MH, RH mouthwash	Plaque, gingival indices	Significant reductions in clinical parameters; CHX more effective; MH ≈ RH
Mathai et al. (2017) [19]	Honey + herbal extracts	*S. mutans*	Limited effect alone; enhanced antibacterial activity in combination (e.g., garlic + lemon)
Jain et al. (2022) [20]	MH mouthwash	*S. mutans*, *Lactobacillus acidophilus*	Significant bacterial reduction; efficacy comparable to CHX
Aparna et al. (2012) [21]	Honey mouth rinse	Oral bacteria; plaque (in vivo model)	Antibacterial activity in vitro and reduced plaque regrowth in vivo; CHX showed greater efficacy

## Conclusions

Honey demonstrates consistent antibacterial and antibiofilm activity against key cariogenic bacteria in vitro, effectively inhibiting bacterial growth and early biofilm formation. However, clinical evidence remains limited and heterogeneous, with variable effects on cariogenic bacteria, plaque accumulation, and oral health outcomes. The overall efficacy of honey appears to be strongly influenced by its botanical origin, physicochemical properties, and formulation, as well as by study design and clinical conditions. While honey exhibits promising antimicrobial properties, its impact on enamel integrity remains uncertain, with conflicting in vitro findings highlighting both potential protective and demineralizing effects under different conditions. Therefore, although honey may represent a valuable adjunct in oral healthcare, it cannot replace established preventive strategies such as mechanical plaque control and conventional chemical agents. Future well-designed, long-term randomized controlled trials using standardized honey formulations are required to clarify its clinical effectiveness and define evidence-based guidelines for its use in dentistry.
